# Fit for purpose? Analysis of the relationship between skull, beak shape and feeding ecology in Psittaciformes

**DOI:** 10.1111/joa.70063

**Published:** 2025-11-04

**Authors:** Shannon L. Harrison, Gregory P. Sutton, D. Charles Deeming

**Affiliations:** ^1^ Joseph Banks Laboratories, School of Natural Sciences University of Lincoln Lincoln UK

**Keywords:** cranium, geometric morphometrics, mandible, maxilla, Psittaciformes; skull shape

## Abstract

Psittaciformes have a broad distribution across the southern hemisphere, and this wide ecological range is coupled with high levels of morphological diversity, particularly in the skull and beak structure, which has been previously linked to diet and body size. This paper studies how the beak and cranial shape vary in relation to predominant diet across a broad taxonomic range of parrots, using 2D geometric morphometric techniques and regression analyses. The data suggest that whilst there are some levels of significance between diet and beak shape, body mass was a much stronger co‐variate. This indicated that skull morphology is more likely explained by parrot body mass and that whilst diet may partially explain beak shape in parrots when compared to other avian groups, it is not determining beak shape within the clade. Further study into other factors related to beak morphology, such as bite force, may prove key in explaining the evolutionary forces shaping the skull in parrots and other birds.

## Graphical abstract

Psittaciformes exhibit high levels of morphological diversity, particularly in skull and beak structure, previously linked to diet and body size. Although there were some levels of significance between diet and beak shape, body mass was a much stronger co‐variate. Diet is not determining beak shape within the clade. Further study into other factors related to beak morphology, such as bite force, may prove key in explaining the evolutionary forces shaping the skull in parrots and other birds.

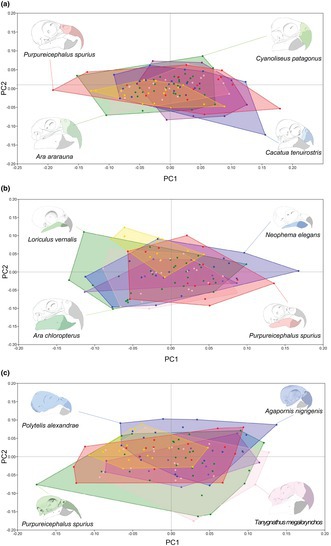

## 1 INTRODUCTION

Birds exhibit a wide range of morphologies, varying in body size, plumage colour, foot shape, etc. (Felice et al., 2019; Sheard et al., 2020; Stoddard & Prum, 2011). One widely studied element of their morphology is the shape of their beaks, particularly in relation to diet (Bhullar et al., 2016; Cooney et al., 2017; Young et al., 2017). A famous example is the beak diversity of Darwin's finches that corresponds to dietary preference (Abzhanov, 2010), but the relationship between beak morphology and diet in other birds continues to be studied (Abzhanov et al., 2004; Grant, 1986; Herrel et al., 2005; Navalón et al., 2019; van der Meij & Bout, 2008). In general, it has been concluded that the feeding ecology of a bird is reflected in its beak shape (Bright et al., 2019; Van Wassenbergh & Baeckens, 2019).

Interestingly, Cooney et al. (2017) found that the evolution of bill shape in modern birds was more rapid towards the beginning of the group's initial radiation; but thereafter, there was generally little evolution of morphological difference within clades. This suggests that beak shape is biologically constrained within closely related taxa and there are a limited number of morphological changes that can occur (Ruta et al., 2013). Therefore, differences between clades are more of a reflection of the initial effects of their ecological expansion rather than being driven by subsequent ecological influence, such as variation in diet.

Beak shape is considered to be advantageous for given diet types because some shapes seem to correlate with certain diets. For example, birds of prey (Falconiformes and Accipitriformes) have sharp, hooked, asymmetrical beaks, which aid in capturing and processing their prey (Sustaita, 2008; Bright et al., 2016). By contrast, waterfowl (Anseriformes) usually possess long and thin more symmetrical beaks which improve their ability to sift through the water for invertebrates and aquatic vegetation (Li & Clarke, 2016), and feeding ecology seems to be the main driver of the evolution of beak shape in waterfowl (Olsen, 2017). However, it is possible that beak shape is indicative of broader feeding ecology rather than being predominantly driven by specific diets. In support of this idea, Navalón et al. (2019) reported that diet only explained about 12% of the variation in maxilla shape for modern birds, leading to the suggestion that a wide array of factors, such as how the beak is used during feeding, could contribute to its shape. Similarly, Bright et al. (2016) found in raptors that the variation in the shape of the beak and skull was mainly explained by body size rather than dietary differences. This finding was supported by Felice et al. (2019) who used a sample of over 350 species of birds to demonstrate that, whilst cranial shape was somewhat related to dietary ecology, the relationship was relatively weak.

Psittaciformes exhibit a wide range in size, ranging from the largest, the Kākāpō (*Strigops habroptilus*) weighing 2000 g to the 12 g Yellow‐capped pygmy parrot (*Micropsitta keiensis*) (Dunning, 2008). Parrots possess a wide range in diets with many feeding on a variety of plant matter, such as flowers, leaves and bark (Gilardi & Toft, 2012), whereas others have a more generalist diet. However, a predominantly plant‐based generalist diet may be more reflective of resource availability, as most species seem to have a preferred diet (Péron & Grosset, 2014). For example, the Salmon‐crested cockatoo (*Cacatua moluccensis*) mainly feeds on fruit and seeds, whilst the Iris lorikeet (*Psitteuteles iris*) feeds predominantly on nectar (Wilman et al., 2014). Despite this, the parrot beak is superficially similar amongst most species with the maxilla typically being distally curved and more robust towards the proximal region, and the mandible being smaller in size (Bright et al., 2019; Tokita, 2003). The overall superficial similarity in beak morphology may also be due to roles that the beak has other than feeding (Navalón et al., 2019), for example many parrots exhibit a locomotive behaviour called ‘beakiation’ (Dickinson et al., 2024) in which the beak is used for grasping thereby serving as a pseudo limb in climbing. However, a few species, for example, the Slender‐billed parakeet (*Enicognathus leptorhynchus*) and Kea (*Nestor notabilis*), have distinctive beaks which, whilst still curving downwards, are long and thin (Juniper & Parr, 1998).

Despite their distinctive beak shape and varied diets, Psittaciformes have been relatively poorly studied regarding the relationship between variability in beak shape and feeding ecology. Many studies have focused on shape changes within birds of prey (Falconiformes and Accipitriformes), and passerines of the Fringillidae and Paridae (Abzhanov et al., 2006; Sustaita & Hertel, 2010; Shao et al., 2016; Soons et al., 2015; Felice & Goswami, 2018). However, Bright et al. (2019) showed that the shape of the skull in parrots was highly dependent on the effects of evolutionary allometry and integration of the cranial elements with diet only accounting for less than 3% of beak shape variation. Similarly, phylogeny predominantly explained the variation in beak shape in an unrelated group of passerines, the cowbirds (Icteridae; Gómez & Lois‐Milevicich, 2021). It would seem, therefore, that the evolution of beak morphology and shape is predisposed to follow a given evolutionary trajectory and may not necessarily be affected by dietary ecology (Cooney et al., 2017; Yusuf et al., 2020).

Following their initial niche occupation after the rapid expansion of the avian lineage, avian skull evolution appears to follow a given shape trajectory, with different families displaying different morphs (Bright et al., 2016; Goswami et al., 2014). This process has led to high levels of integration between the cranium and the maxilla and to date studies have mainly focused on the upper portion of the avian skull (Bardwell et al., 2001; Bright et al., 2016; Kawabe et al., 2013; Navalón et al., 2019). The study described here builds upon the study by Bright et al. (2019) by using geometric morphometrics to quantify shape variability in the skull and the beak in Psittaciformes. Here, the parrot skull was split into three separate components, namely the mandible, maxilla, and cranium and maxilla combined, to allow for more detailed analyses into how different aspects of the skull may be related to diet.

What scaling relationships would one expect for the morphology of a psittaciform beak? If we assume that the geometry of the beak evolved to maintain a constant stress (*σ*) on the bones in the jaw, we can develop a quantitative hypothesis for how we predict beak morphology will change with animal size. For a bird of an arbitrary size, the stress (*σ*) in the bones at a location a distance, *x*, away from the center of the bone is equal to the bending moment (*M*) multiplied by *x* and divided by the product of the area moment of inertia (*I*) and the Young's modulus (*E*), represented in Equation (1) (Popov, 1990):

(1) $$\sigma =\frac{\mathrm{Mx}}{\mathrm{EI}}$$

Assuming isometric growth, the bending moment, *M*, will scale as Length^3^ (muscle force, scaling by length^2^ (Zajac, 1989), multiplied by moment arm, which scales by length^1^), the distance from the centre of the bone, *x*, will scale by length, and the area moment of inertia, *I*, will scale by length^4^ (Popov, 1990). The scaling of the stress, as an animal increases isometrically in size, thus results in Equation (2).

(2) $$\sigma =\frac{L^{3}L}{L^{4}}=\text{Constant}$$

Which predicts that, to keep bone stress constant as a function of size, the morphology of psittaciform beaks should scale isometrically, with shape being independent of animal mass. For Anseriformes, this has been shown to be the case, with beak shape not being significantly affected by bird mass but instead being driven primarily by diet (Olsen, 2017). For Psittaciformes, however, assuming isometric increases of muscle force (proportional to *L* ^2^), may not be correct, as measurements of jaw muscle mass (Harrison et al., 2025) have shown that larger parrot species have proportionally larger jaw muscles, with the muscle mass scaling with length^4^ (or with mass^1.34^). Consequently, as parrot species get larger, their proportionally larger jaw muscles cause their muscle force to scale more closely to length^3^ (alternatively, mass^1^) than to an isometric length^2^; more precisely, Harrison et al. (2025) found that the force scaled to length^2.93^ (or with mass^0.978^). If force scales to length^3^, then Equation (1) predicts that, as psittaciforms get larger, their jaws must get proportionally shorter (reducing the moment arm) or thicker (increasing the area moment of inertia), or both, such that the stress remains constant. This underpins our hypothesis that the morphology of psittaciform beaks will not scale isometrically – instead larger psittaciforms will have relatively shorter and more robust beaks to compensate for the relatively larger forces that the muscles produce.

Geometric morphometrics was used to quantify shape changes in the three separate components of the skull to test the hypothesis that predominant diet type is related to the shape of the component parts of the skull and beak in parrots. In particular, we hypothesise that the shape of the bones will reflect the muscle forces exerted upon them during the processing of the food items with those species (Harrison et al., 2025), such as the Hyacinth macaw (*Anodorhynchus hyacinthinus*), which typically consume hard nuts and fruits, possessing more robust upper and lower beaks compared with species, such as the Rainbow lorikeet (*Trichoglossus haematodus*), which rely much more on nectar in their diet (Juniper & Parr, 1998).

## 2 METHODS

### 2.1 Sources of digital images

Two‐dimensional digital images of parrot skulls were taken at the Natural History Museum, Tring. Images were recorded using a digital camera (Nikon dslr), placed at roughly 30 cm distance from the specimen in lateral aspect, with the specimen's beak facing towards the right. A linear scale was included in each photograph. Two images of each skull were obtained. The first had the mandible in place, whereas in the second the mandible was absent thereby exposing the lateral surfaces of the palatine bone. Additional images of parrot skulls with mandibles in place were downloaded from www.Skullsite.com, which is a collection of skulls that is managed and digitised by The Experimental Zoology Group of Wageningen University, The Netherlands. Specimens retrieved from Skullsite were only used if they fitted the criteria for shape analysis, that is, rightwards facing and image taken from a lateral perspective with the rhamphotheca present. The skulls had to be of similar orientation to ensure that the points that were being sampled were comparable between specimens. These images were edited to include a scale bar derived from the accompanying length data reported on the website. The length of the skull associated with each set of images was confirmed as being the basicranial measurement from the back of the cranium to the outermost point of the beak taken from dorsal view (Jan Jansen, Wageningen University, personal communication, 2020).

In total, skulls of 67 species were available from the NHM Tring collection, 37 species were available from www.skullsite.com, and 7 species were prepared at Lincoln from birds dissected for a study of bite force in parrots (Harrison et al., 2025; for a list of species included in the study see Table S1). A lateral view of the species without the mandible was not available from http://skullsite.com. In addition, due to the arrangement of the skulls in the photographs, there were fewer samples for the mandibles because closure of the jaws obscured the tip of the mandible in some cases, such as in the case of *Anodorhynchus hyacinthinus* from the Tring sample site, although this species was also available from Skullsite. If there was not a suitable image of a given species across the three sample views, it was not included in the analysis.

### 2.2 Digital landmarking of skull shape

**FIGURE 1:**
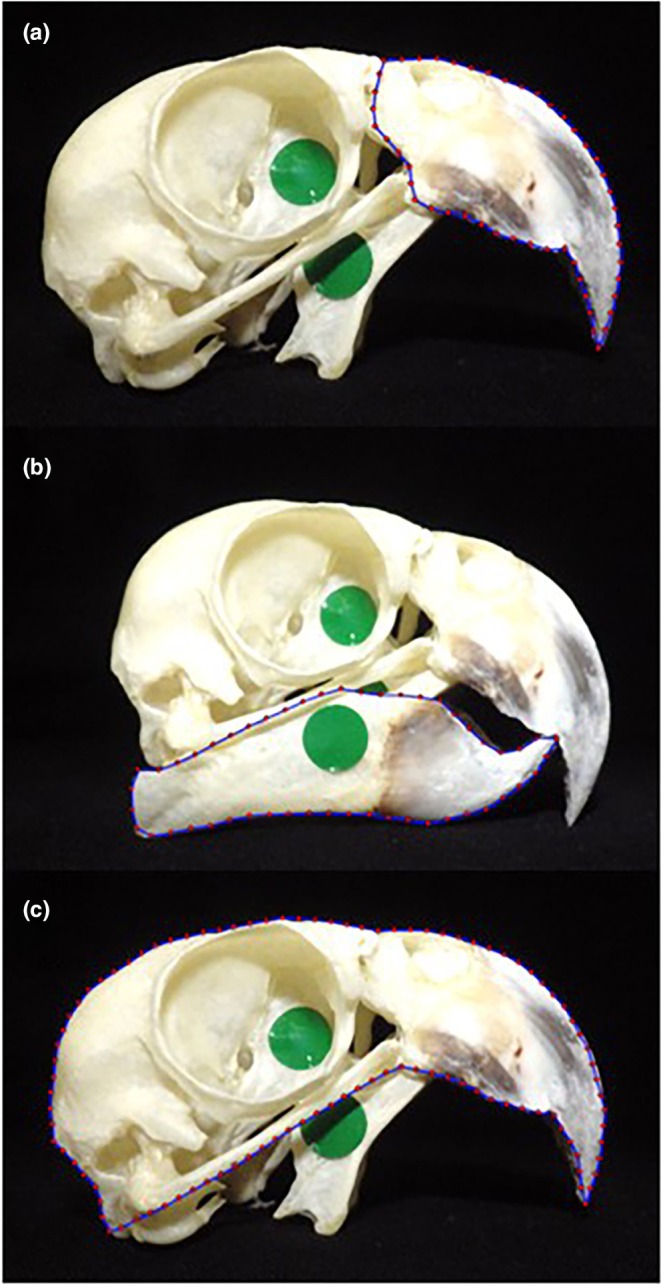
Landmark placement (red dots and blue line) for the three separate components of the skull of *Amazona auropalliata*. a = maxilla shape, b = mandible shape and c = cranium and maxilla shape.

Digital landmarks were placed within TPSDig2 software (Rohlf, 2006) along the outermost edges of three different components of the skulls: the maxilla, mandible and whole cranium and maxilla. The component shapes of the mandible and maxilla were defined using 50 equally spaced non‐sliding semi‐landmarks (Gunz & Mitteroecker, 2013; Rohlf, 2006), positioned along the edge of the given component. Landmarks were used to outline the cranium and maxilla combined (Figure 1). This method was chosen to define beak shape because it provides more information on the shape changes of aspects of the component (e.g., the rostral edge boundary), which are not necessarily discrete structures. Use of semi‐landmarks also maximised the level of ‘semi‐landmark homology’ in the absence of distinct homologous structures (Gunz & Mitteroecker, 2013), as is commonly required in geometric morphometric measurements (Klingenberg, 2011). However, it should be noted that for each set of shape analyses, the tip of the maxilla/mandible acted as the start point for the 50 semi‐landmarks, acting as an anchor point between the different species photographs. This meant that in combination with the even spacing, the points along the curves were all directly comparable with their counterparts on different specimens.

For the upper beak, otherwise known as the maxillary rostrum (and referred to as maxilla hereafter), the semi‐landmarks always started at the distal tip of the maxilla and were placed in an anti‐clockwise direction to trace its outline following upwards towards the cranio‐facial hinge (Figure 1a). At the cranio‐facial hinge the outline followed the edge of the maxilla towards the point at which it attached to the jugal bar. The outline continued along the tomial edge of the rhampotheca back towards the beak tip (Figure 1a).

For the mandible, the first landmark was always placed at the distal tip of the rhampotheca, with subsequent points being placed along the dorsal edge of the rhampotheca and bone in an anti‐clockwise direction (Figure 1b). The landmarks traced the angular regions of the mandible, circling back towards the distal tip of the rhampotheca.

The outline of the cranium and maxilla region comprised both the maxilla and the cranium. The first landmark was placed at the distal tip of the maxilla, with subsequent points running in an anti‐clockwise fashion in a caudal direction (Figure 1c) and continuing past the cranial hinge, over and around the cranial bones, eventually reaching the quadrate. Due to inter‐specific differences in cranium and maxilla shape, that is, the degree of fusion of bony elements around the orbit, the anterior edge of the jugal bar was used as a reference point when tracing the outline back towards the tip of the maxilla. The rest of the maxilla was then defined by landmarks placed along the tomial edge back towards the beak tip.

Initial positioning of landmarks was visually checked and positions of individual landmarks were revised to ensure that they accurately defined the perimeter of the structures. The semi‐landmarks were then re‐sampled by equal length until the outline was deemed as accurate as possible. To minimise inter‐observer error, SLH performed all placements of landmarks.

### 2.3 Morphometric analysis

The x‐y coordinates for each skull were collated and imported into MorphoJ (Klingenberg, 2011) for analyses. A Procrustes fit was performed on the data set to remove the effects of size and rotational differences (Mitteroecker & Gunz, 2009) before being transformed into a co‐variance matrix, which was subjected to principal component analysis (Jollife & Cadima, 2016). From these analyses, graphs with smooth wire frame outlines were generated to inform the various shape changes across the principal components (±1SD) which represented the highest percentage variance.

### 2.4 Dietary classification

**FIGURE 2:**
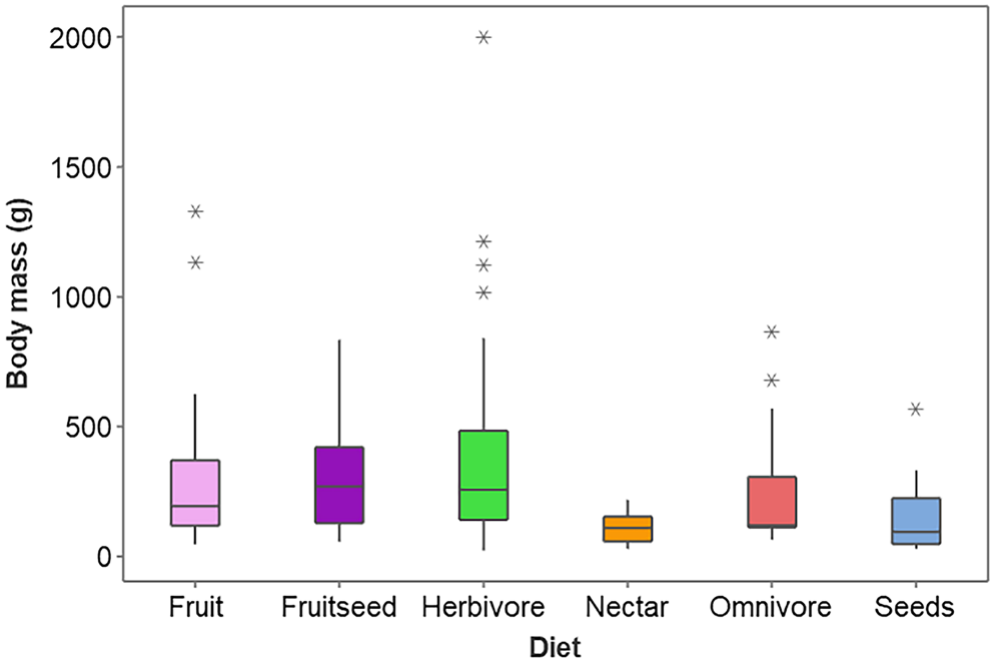
The median, interquartile ranges and range of body masses for parrot species classified according to diet.

Dietary classification was based on the data set compiled by Wilman et al. (2014) that documents the percentage of the diet of particular food items: invertebrates, nectar, plants, seeds and fruit. However, closer examination of the data used in this study suggested that the three criteria Wilman et al. (2014) defined for parrots were not representative. For instance, Wilman et al. (2014) classified the diet into specific categories such as ‘Fruitnectar’ for the Yellow‐streaked lory (*Chalcopsitta sintillata*), despite the percentage composition of the diet being 100% nectar. Some other species were categorised too specifically, despite there being three or more separate food items found within their diet. For example, the Cape parrot (*Poicephalus robustus*) was classified as being ‘Fruitnectar’ despite seeds making up 40% of their diet. Therefore, the species' diet was reclassified based on three elements. First, the presence of invertebrates within the diet, regardless of the percentage composition for plant material, classified the species as an omnivore. If animal material was absent in the diet, then the highest percentage value in the composition of the food item was taken as the species' predominant diet. However, if there was a 10% difference between two food items the species was classified as a 50/50 food type, that is, ‘fruitseed’. Finally, if there were three or more plant‐based food items then the diet was classified as a ‘Herbivore’. In total, six diet categories, that is, omnivore, nectar, herbivore, seed, fruitseed and fruit, were recognised (shown for each species in Table S1). Digital images were not always available for each of the species used for the three series of landmarks and sample sizes for each dietary category are shown in Table 1. The median body masses of the species represented in each of these diet categories were significantly affected by the diet category with nectar‐feeding and seed‐eating species being smallest and herbivores and fruitseed species being the largest (Figure 2; Kruskal–Wallis test: *H* = 19.91, DF = 5, *p* = 0.001).

**TABLE 1:** Numbers of parrot species represented in analyses testing for the effect of dietary classification (as defined) on skull or beak shape.

Diet category	General composition	Mandible	Upper beak	Skull
Fruit	>70% Fruit in diet	20	21	21
Fruitseed	50:50 Fruitseed	9	12	12
Herbivore	Three or more plant‐based diet types	31	32	32
Nectar	>70% Nectar in diet	12	14	14
Omnivore	Animal and plant materials in diet	16	18	18
Seed	>70% Seed in diet	15	16	16
	Total species represented	103	113	113

*Note*: See Table S1 for diet classifications for individual species.

### 2.5 Statistical analysis

Initial analysis involved a data set that consisted of the values for PC1, PC2 and PC3 for each given shape (before phylogenetic correction) for each species plus its dietary classification. These data were loaded into PAST version 4.0 (Hammer et al., 2001) and data for each species were colour‐coded according to the diet type. To initially test the effect of group (in this case diet type) on our PC1 and PC2 variables a one‐way PERMANOVA was run with 10,000 permutations using a Euclidean similarity index.

Body mass values were log_10_ transformed prior to analysis to obtain residuals that were normally distributed and homoscedastic. In order to account for the effects of phylogenetic relatedness, analysis was performed in R (version 4.0.3; R Development Core Team, 2021) using the statistical package ‘*geomorph’* (Adams et al., 2025). Linear phylogenetically generalised least squares (PGLS) regressions were used to test the effects of diet on the different shapes, using the function *procD.pgls* in *geomorph*. These regressions were used to test the hypotheses that there would be significant differences in skull shape according to diet type and body mass. The regressions for each skull component were as follows (shape ~log body mass */+ diet), in which 5000 permutations were run for each model. For each individual species, the extracted landmark coordinates underwent Procrustes superimposition (using the package *gpagen*) prior to the PGLS regression analysis and as such ‘shape’ refers to the entire aligned landmark data (Adams et al., 2025). By aligning the shape coordinates prior to PGLS analysis it means that the shape is considered as a whole unit opposed to being split into principal components, which allows for a more robust analysis of the relationship between shape and diet (Bright et al., 2019). In instances whereby the interaction term was not significant, the model was simplified and repeated without the interaction term. Due to the differing number of species in each shape data set, two separate time‐calibrated trees were downloaded (see Figure S2a,b) from http://birdtree.org (Jetz et al., 2012) and loaded into the R environment using the package ‘*phytool*s’ (Revell, 2012).

## 3 RESULTS

### 3.1 Maxilla shape

**FIGURE 3:**
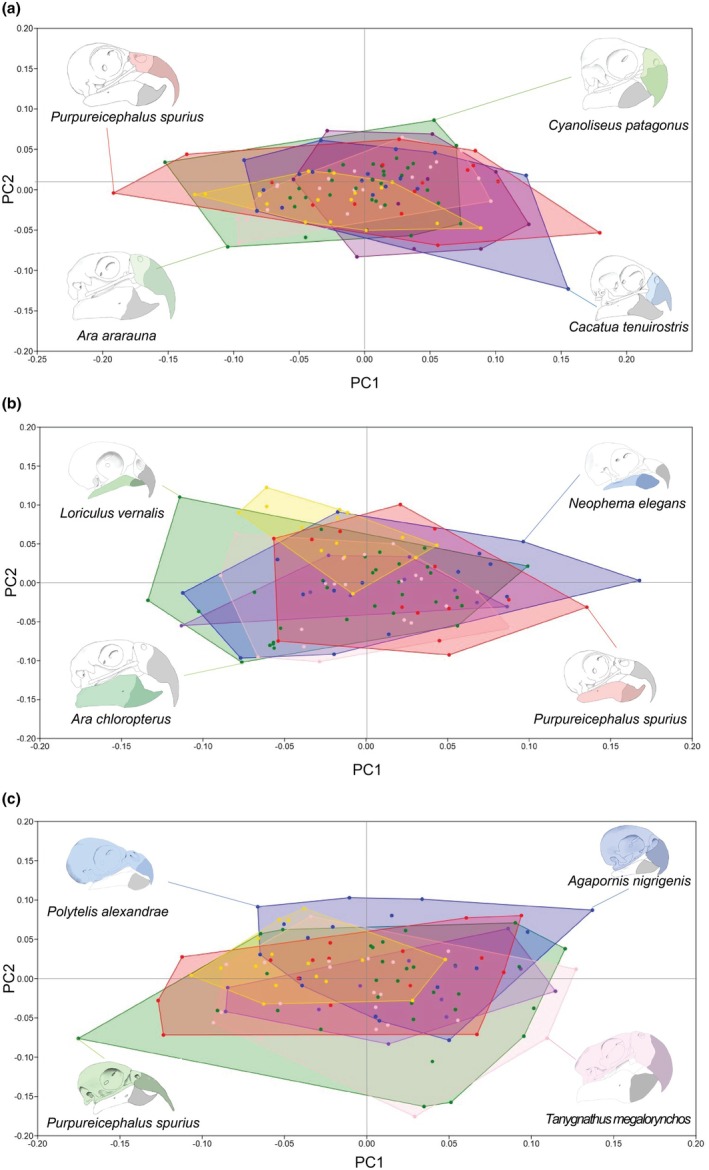
Convex hulls plot of the extremes for PC1 versus PC2 plot of the maxilla shape (a), mandible shape (b) and cranium and maxilla shape (c) according to dietary types; yellow = nectar, red = omnivore, green = herbivore, blue = seed, purple = fruitseed and pink = fruit.

The maxilla experienced the most shape variation within the first three principal components which explained around 50%, 16% and 15%, respectively, of the shape variation (Figure S1). Variation along PC1 represented the greatest difference in tomium depth with more positive PC1 values being associated with a maxilla which is broad anteroventrally (Figure 3a). More negative PC1 values were associated with a thin maxilla which exhibited very little difference in depth between the tomium and the commissure (Figure 3a). The greatest shape changes along PC2 seemed to be around the tomium; although there were also some changes around the dorsal point of the beak with more positive PC2 values associated with a narrowing of the distal maxilla to produce a more notched effect in the tomium (Figure 3a). As the PC2 value reduced and became more negative this notch was noticeably reduced (Figure 3a).

**FIGURE 4:**
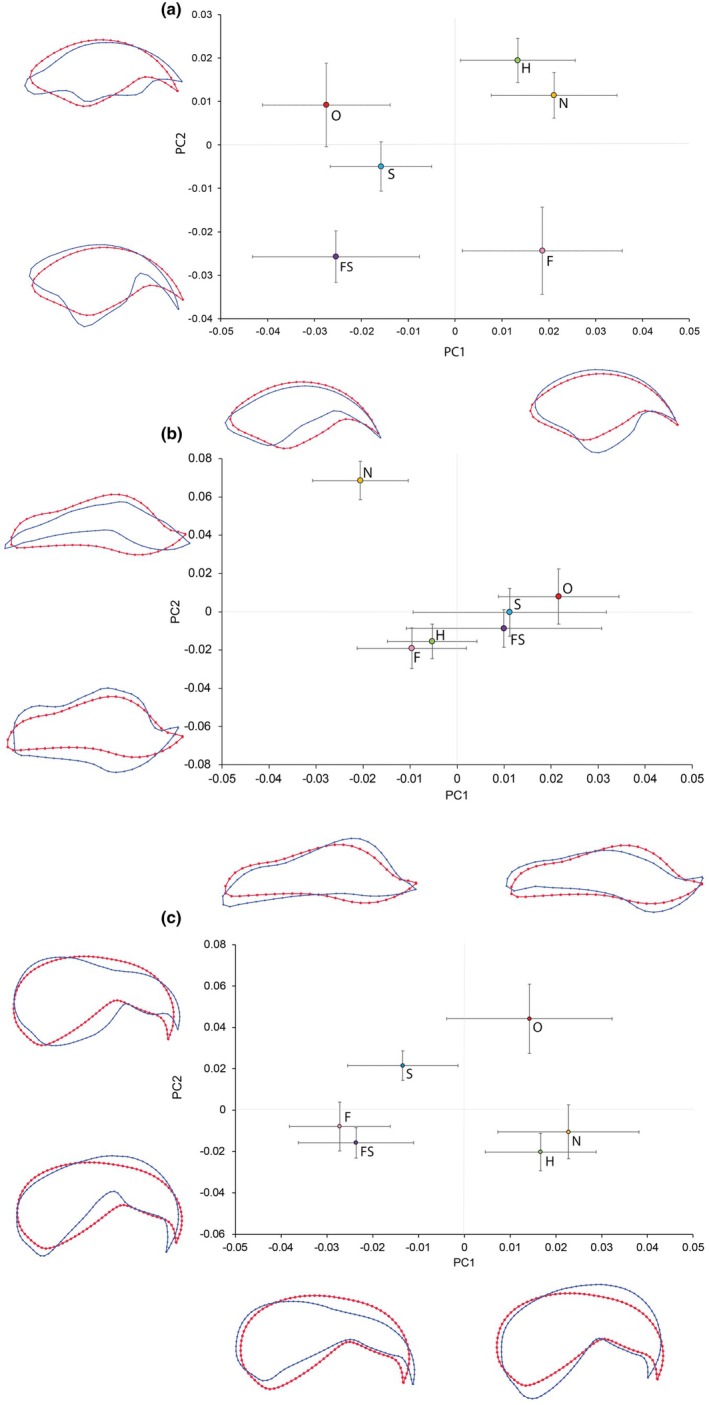
Mean dietary (N = nectar, O = omnivore, H = herbivore, S = seed, FS = fruitseed and F = fruit.) PC scores for of the maxilla shape (a), mandible shape (b) and cranium and maxilla shape (c). Wireframes produced using MorphoJ for PC1 and PC2 of the cranial components. Each wireframe shows the mean shape (red dotted outline) and its respective shape deviation (blue dotted outline) along a PC axes.

When PC scores for each species were plotted within the same morphospace according to dietary category there was considerable overlap between the convex hulls for all diet types (Figure 3a). Data for herbivores, omnivores, fruit eaters and seed eaters covered the largest morphospace range, particularly for PC1, and almost entirely occupied the same morphospace as nectar and fruitseed feeders. These two more specialist diet types appeared to have a slightly reduced morphospace occupation with clustered points. A slightly more exaggerated partition was found when these PC scores were averaged and plotted against one another according to diet (Figure 4a). Generally, there was a grouping of the average PC scores, with the more generalist diets grouped together with PC values in the mid‐range with each diet type experiencing some overlap. Herbivore and nectar feeders were found in the same quadrants, whilst fruit and fruitseed feeders differed along PC1. Nectar feeders seemed to possess maxillae that were more streamlined in shape occupying the more negative segments of the shared morphospace, lacking the defined tomium and deep beak as found in omnivorous species which had more positive PC1 values. PERMANOVA analysis performed to test the effects of diet on PC1 and PC2 showed no significant interaction between shape and diet (*F*_1,5_ = 1.092, *p* = 0.370). For all diet types there was only one instance of a significant difference in shape in the pairwise analysis. Fruitseed and nectar feeders were significantly different (*p* = 0.015); there was no significant difference between the shape of the other diet types.

**FIGURE 5:**
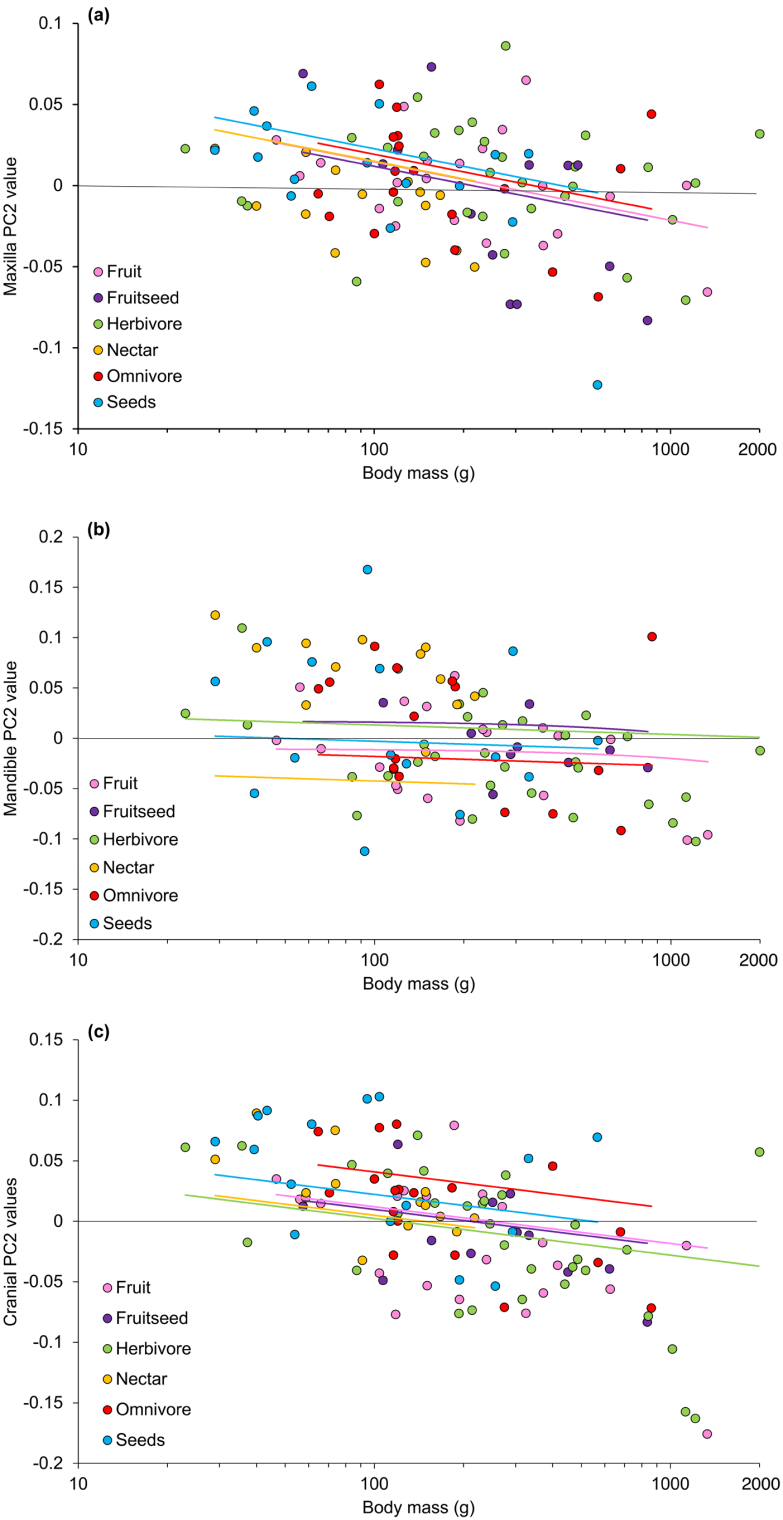
Relationship between PC2 values and average body mass (Dunning Jr, 2008) between the different dietary categories for the maxilla (a), mandible (b) and cranial and maxilla (c). Trendlines are derived from PGLS regressions generated in R.

Phylogenetically controlled linear modelling ANCOVA of maxilla shape against log_10_ body mass and diet revealed that there was no significant interaction between shape and diet type whilst controlling for body mass (*F*_5,112_ = 1.53, *p* = 0.157). When the interaction term was removed, both diet and log body mass exhibited significant negative relationships with shape (Table 2; Figure 5a). Along PC2 the greatest shape change (Figure 5a) is mainly concentrated around the tomium, with more positive PC2 values being associated with a more narrowed distal maxilla and consequently a noticeably more notched tomial edge. More negative PC2 values represent a more smoothed, less noticeable tomium. A significant negative relationship was found between PC2 and log body mass, suggesting that as parrots get larger, their maxillae have a smoother tomial edge. Diet explained around 11% of shape variation within the mandible, and log_10_ body mass explained 8% of variation in shape.

**TABLE 2:** Results (*F*‐values and associated *p*‐values) from phylogenetically controlled general linear modelling to test for the effect of diet on PC1, PC2 and PC3 values whilst controlling for log_10_ body mass as a co‐variate for the (1) maxilla, (2) mandible and (3) cranium and maxilla.

	*F*‐value (*p*‐value)	*R* ^2^
Maxilla		
Log_10_ body mass (DF = 1112)	10.03 (0.001)	0.08
Diet (DF = 5112)	2.96 (0.039)	
Mandible		
Log_10_ body mass (DF = 1102)	2.79 (0.034)	0.03
Diet (DF = 5102)	1.11 (0.317)	
Cranium and maxilla		
Log_10_ body mass (DF = 1112)	10.06 (0.003)	0.08
Diet (DF = 5112)	2.79 (0.048)	

*Note*: There was no significant interaction terms between diet and Log_10_ body mass for any of the full models, which were subsequently simplified by removing the interaction term and results from the simplified model are reported.

### 3.2 Mandible shape

PC1 and PC2 both were nearly equivalent in the explanation of variation for mandible shape, representing around 38% and 35%, whereas PC3 explained around 8% of the variation (Figure S1). Amongst the more positive values of PC1 the mandible appeared to experience a shift in shape about the more caudal region of the jaw which becomes more dorsally elongated, whilst the rostral region of the beak appeared to be more outwardly curved. Towards the more negative PC1 values, the mandible becomes more streamlined (generally thinner), with the rostral curve being relatively reduced but the lower aspect of the jaw was relatively more curved. PC2 represented changes in the thickness of the mandible, moving from a robust, deep shape for negative values to a more gracile shape which thins out at the articular region for positive values (Figure 3b). A similar positive–negative pattern was found within PC2 whereby the comparably larger mandible shapes are represented by negative PC values and vice versa (Figure 3b).

When average values were plotted for PC1 and PC2 (Figure 4b) most of the dietary categories exhibited variation in PC1 but very little variation in PC2. The exception was the mean value for PC2 for nectar‐feeding species, which occupied a much more positive value than the means for the other dietary categories (Figure 4b). One‐way PERMANOVA results indicated that there was a highly significant relationship between PC1, PC2 and diet (*F*_1,5_ = 3.41, *p* < 0.001). This high significance was a result of the shape of the mandible in nectar‐feeding species, with each diet type being solely significantly different from nectar feeders.

Phylogenetically controlled linear modelling ANCOVA of mandible shape against log_10_ body mass and diet revealed that, unlike in the maxilla, diet was not a significant co‐variate affecting mandible shape (Table 2). Similarly, there was no significant interaction between shape and diet type whilst controlling for body mass (*F*_5,102_ = 0.89, *p* = 0.529). However, log_10_ body mass had a significant negative effect on shape by body mass (Table 2), this is particularly evident when viewing PC2 shape change in tandem with log_10_ body mass (Figure 5b), whereby as body mass increases, PC2 values tend towards a decrease representing the transition between a dorsoventrally flattened beak and the more robust form of the heavier species such as the macaws. Diet explained around 5% of shape variation within the mandible, and log_10_ body mass explained 5% of variation in shape, suggesting that mandible shape is equally affected by both diet and body mass.

### 3.3 Cranium and maxilla shape

PC1 and PC2 accounted for around 44% and 34%, respectively, of the variation in shape change in the cranium and maxilla. Changes along PC1 represented a shift in shape from a cranium that is more rounded and streamlined to more robust shapes with a deep maxilla and more cuboidal skull shapes. The majority of the variation seemed to be around the lowest point of the cranium and along the zone around the cranial hinge. Species with more negative PC1 values were associated with a shape which is more streamlined and spheroid whilst those on the opposite end of the axis comprised cranium and maxilla shapes that were bulkier, extending outwards from both the cranial hinge and articular region (Figure 3c). By contrast, changes within PC2 were associated with further narrowing of the hinge region and elongating ventrally around the mid region of the skull. More positive PC2 scores were representative of a more slender form which had shorter maxillae whilst species found occupying more negative PC2 values within the morphospace were more rounded at the most cranial aspect of the skull.

Convex hulls for cranium and maxilla shape according to diet exhibited a general occupation of the area surrounding the centroid value (Figure 3c). Interestingly seed and herbivore feeders had opposing positions in morphospace for PC1. Nectar feeders maintain the pattern of being the most gracile in shape with the convex hull for this group being the smallest. Omnivores also seemed to occupy the most generalist morphospace with values which spaced in all four quadrants almost equally.

Mean PC scores for each dietary type showed that skull shape varied depending on the predominant diet (Figure 4c). Nectar‐feeding birds occupied a more positive PC1 range in comparison to other diet types, with skulls which conform to a more rounded cranial element with little definition between the maxilla and cranium. The omnivores and herbivores were also found within the positive PC1 values (although less positive than the nectar feeders). One‐way PERMANOVA results indicated that there was a highly significant relationship between PC1, PC2 and diet (*F*_1,5_ = 3.32, *p* < 0.001). As with the mandible results, nectar feeders were significantly different in cranium and maxilla shape when compared to the other diet types.

Phylogenetically controlled linear modelling ANCOVA of the shape of the cranium and maxilla against log_10_ body mass and diet revealed that whilst individually log_10_ body mass and diet both had a significant effect on cranial shape (Table 2; Figure 5c) there was no significant interaction between the two co‐variates (*F*_5,112_ = 1.41, *p* = 0.195). Much like in the maxilla, there was a (albeit less pronounced) negative relationship between cranial shape and PC2. The larger parrots typically inhabited lower PC2 values within the morphospace, meaning that their skulls tend to become more curved at the cranial region, with a more robust intercranial hinge. Diet explained around 11% of shape variation within the cranium, and log_10_ body mass explained 8% of variation in shape. Consequently, it appears that across the three different skull regions, diet and body size are both independently driving changes in beak shape.

### 3.4 Mechanical perspectives of beak shape

In the introduction it was suggested that from a mechanical perspective the strong bite forces of parrots (Harrison et al., 2025) mean that dimensions of the skull and mandible should not scale isometrically with body mass (BM). However, in parrots, the results above showed a significant negative relationship between body mass and mandible shape. A preliminary follow‐on investigation recorded the mandible length (ML in mm) and mandible height (MH in mm) for a subset of parrot species in the present study that had been photographed at the NHM. Phylogenetically controlled analysis showed that mandible length scaled with positive allometry with body mass (ML = 5.2*BM^0.372^, *F*_1,51_ = 499.4, *p* < 0.0001, *R* ^2^ = 0.907); a one‐sample t‐test of the slope against the expected slope of 0.333 was significant (t_51_ = 2.53, *p* = 0.014). This effect was even greater for the relationship between mandible height and body mass (MH = 0.7*BM^0.504^, *F*_1,51_ = 204.2, *p* < 0.0001, *R* ^2^ = 0.800; t_51_ = 4.94, *p* < 0.0001).

By contrast, the Anseriformes exhibit relatively different specialised feeding modes: filter‐feeding with a flat, mediolaterally expanded bill; cropping or grazing vegetation with a dorsoventrally expanded bill; and a narrow bill used in grasping aquatic invertebrates and fish (Li & Clarke, 2016). Whilst bite force values are unavailable for waterfowl (Deeming et al., 2022), bill shape has been shown to correlate with diet instead of body size (Olsen, 2017). Moreover, for any given body mass jaw musculature mass, which correlates strongly with bite force (Deeming et al., 2022), is much lower in Anseriformes than in Psittaciformes (Deeming et al., 2024). This suggests that mandible dimensions should scale isometrically in Anseriformes (not explored by Olsen, 2017). To explore this, in a preliminary study data for 37 species of waterfowl were available in Ellrott and Schmitz (2010) and body masses from Dunning (2008). Phylogenetically controlled linear analysis revealed significant relationships between body mass and mandible length (ML = 6.3*BM^0.368^, *F*_1,35_ = 104.5, *p* < 0.0001, *R* ^2^ = 0.749) and with mandible height (MH = 1.1*BM^0.308^, *F*_1,35_ = 162.8, *p* < 0.0001, *R* ^2^ = 0.823). The slope of these relationships did not differ from the expected isometric slope of 0.33 (predicted slope of 0.333; t_35_ = 1.04, *p* = 0.305, t_35_ = −0.92, *p* = 0.365, respectively) demonstrating proportional scaling of mandible size and body mass.

## 4 DISCUSSION

The results presented here suggest that in Psittaciformes, the shape of the skull, the maxilla and the mandible were all significantly affected by body mass. Smaller species possessed more similarly shaped skulls compared with larger species and vice versa. Additionally, there were significant effects on the cranium and maxilla shape (but not mandible) in response to different diet types although the extent to which these differences were explained by diet type was relatively low (a maximum of 11%). As predicted, as Psittaciformes get larger, their beaks get proportionally shorter and thicker, consistent with the primary driver of this beak shape being the increased mechanical demands on the bones caused by their positively allometric increases in bite force.

The main influencing factor of beak shape in Psittaciformes is thus body size due to the increasing forces caused by their proportionally larger beak muscles (Deeming et al., 2022), with diet being a relatively low predictor of beak shape. This is in direct contrast with Anseriformes, in which beak shape is driven primarily by diet, and not at all by animal size (Olsen, 2017). This result indicates very different evolutionary drivers of beak shape in the two orders. The differing evolutionary pressures on the beaks in ducks and parrots thus demonstrate very different scaling laws for morphology in the different orders of birds.

### 4.1 Beak shape and diet in birds

Parrots have typically been classified as generalist feeders (Benavidez et al., 2018; Bright et al., 2019; Heinsohn et al., 2018). Bright et al. (2019) suggested that the generalist diet stems from their initial range expansions away from Australia into areas where the ecological landscape and climate were relatively temperate, which affected available food items (Schweizer et al., 2011) and was changeable during the Miocene. As a result, species could not afford to be particular with their diet, instead consuming in a more generalist sense (Bright et al., 2019). The classification of diet in the species studied here was derived from Wilman et al. (2014) whereby the overall dietary preference was classified according to the highest percentage food item. The wide range of diet types studied here may have slightly concealed underlying relationships between diet and beak shape because each species was sorted into discrete categories, rather than being classed as a generalist. However, these categories were created as broad as possible to minimise this risk, e.g., use of the term ‘nectar’ opposed to its initial constituents of ‘Fruitnectar’, ‘Nectarplant’ and ‘Nectarmixed’, as seen in Wilman et al. (2014).

Navalón et al. (2019) and Bright et al. (2019) showed that the link between dietary ecology and beak/skull shape in a wide variety of birds explained less than 12% of beak shape variation. However, despite the significant relationships found between shape and diet, the influence on skull shape(s) reported in our study also only explained a maximum of around 8% of beak and cranial variation. Bright et al. (2019) proposed that instead the differences in beak shape were factors of integration and evolutionary allometry, which, whilst not explicitly tested in this study, given the tight association between the morphology of the maxilla and cranium, may explain why both the cranium and maxilla shapes were significantly affected by diet, whilst mandible shape was unaffected.

Cooney et al. (2017) found that at the beginning of the avian expansion, there were high levels of phenotypical disparity between beak shape, meaning that many beak forms were generated between groups. However, after this initial expansion rate, change amongst the clades was relatively stable because many lineages maintained a similar morphological structure in their beaks, slowly accruing minor shape variation, although these differences generally evolved along a similar shape axis. This suggests that there was some form of biological constraint which prevented further lineages from generating new morphologies. Yusuf et al. (2020) demonstrated that changes in coding and non‐coding genes were coupled with the cranio‐facial development of both mammals and birds. It was also suggested that if the basis of phenotypic change is a result of genetic change, populations may experience a lower level of genetic abundance after an extinction event (such as the Cretaceous‐Paleogene event) which reduces the effective population, genetically restricting the population as they re‐diversify.

Navalón et al. (2019) found that in parrots there was one ancestral shift which subsequently caused the rate of shape change to increase rapidly, leading to the notable curved and robust skull shape seen in many extant parrots. The parrot skull is considered to be relatively integrated with its maxilla (Bright et al., 2019), meaning that the two elements are relatively fused to one another, that is, a change in one component will likely be reflected in the other. A similar level of integration was found in raptors with integration of cranium and maxilla; this coupling between the two components accounted for around 80% of shape variation, diet having very little effect on shape (Bright et al., 2016). Additionally, Bright et al. (2016) found that integration of the cranium and maxilla followed similar morphologies across different families, which was interpreted as evidence for an underlying biological constraint which influenced skull shape across lineages.

This highlights the idea that the maxilla is not as independently affected by dietary ecology as initially thought, but rather there are likely a range of both biological and ecological factors that may alter and confine beak shape (Cooney et al., 2017; Yusuf et al., 2020). Whilst the shape of the maxilla may not be solely affected by diet type, the results presented here indicate that there is a definitive link between shape and diet in parrots. Given that the mandible was not significantly influenced by diet, it suggests that the shape of the maxilla is more influenced by diet relative to other areas of the skull. Therefore, this indicates that, despite the potential biological constraints previously reported, beak shape and diet type in parrots are, tentatively, reflective of one another.

In waterfowl (Anseriformes), it has also been found that the shape of the beak was significantly correlated with their diet and feeding ecology, with 64% of the variation in bill shape being explained by the association between diet and beak shape (Olsen, 2017). Interestingly, Cooney et al. (2017) found that Anseriformes had a higher relative rate of beak shape evolution than in other clades and suggested that perhaps the waterfowl's distinctive diet was a major driver of this higher rate of evolution. This association between diet and beak shape in Anseriformes demonstrates how macro‐evolutionary processes, such as development restrictions, are not always representative of every clade and as such continual study of individual groups is important as different patterns of evolution emerge which may conflict with an assumed overall pattern.

### 4.2 Beak shape and body mass

For all elements of the skull shape measured, there was a significant interaction between shape and body mass. Interestingly, despite the lack of significant interaction between shape and diet in the mandible, there was a significant relationship between body mass and mandible shape. This was particularly emphasised when comparing the differences in mandible shape for PC2 and body mass. However, this superficial difference between mandible shape and body mass has been relatively unexplored in birds, with most studies focusing on the maxilla or the cranium (Bright et al., 2016; Mallarino et al., 2012; Soons et al., 2015; Young et al., 2017).

Parrot diets can be considered quite generalist because they feed on a wide variety of plant and/or animal materials (Gilardi & Toft, 2012; Juniper & Parr, 1998) but nectarivory is a specialised diet in parrots (Schweizer et al., 2014). Other nectar‐feeding bird species are generally known for possessing long and thin beaks which are thought to aid in the collection of nectar at the base of flowers (Rico‐Guevara et al., 2019). Observations of the feeding behaviour in Australian honeyeaters (Meliphagidae, Passeriformes) and hummingbirds (Trochilidae, Apodiformes), suggest that beak length is proportionate to the floral lengths, and it has been suggested that the slight curvature of the beak facilitates easier feeding within the flower as it allows the bird to perch on the branch that the flower is on whilst still being able to reach the nectar (Berns & Adams, 2010; Temeles et al., 2009). Perhaps in the case of nectivorous parrots the thinner upper and lower beak aids in feeding behaviour by contributing to an overall thinner feeding apparatus, which is relatively unhindered by its jaw (Yanega & Rubega, 2004). Nectar feeding has been classed as a specialised diet type (Felice et al., 2019) with well‐known nectar feeders, such as the Hawaiian honeycreepers (Fringillidae) being highly adapted to feed on the Hawaiian lobelioids. When these plants were in decline the honeycreepers experienced a shift in bill length to a gradually shorter form that is more reflective of the Ohio tree (*Metrosideros polymorpha*; Smith et al., 1995). Therefore, the apparent association between diet and beak shape in parrots could reflect the fact that the group possesses a relatively high level of variability in dietary preferences between clades. Specialisation towards a given food type, such as nectarivory, has led to significantly different beak shapes. This follows the same trend previously discussed in waterfowl, another group known for taking advantage of an unusual food source (Olsen, 2017). This idea supports the findings of Felice et al. (2019) who demonstrated that granivorous and nectivorous species of birds experience higher rates of cranio‐facial shape evolution than insectivorous or omnivorous birds.

From a mechanical perspective the strong bite forces of parrots mean that dimensions of the skull and mandible should not scale isometrically with body mass, but they did. Mandible height, however, increased with positive allometry (increasing with mass^0.5^) greatly increasing the area moment of inertia of the mandible, as the area moment of inertia is proportional to the square of the mandible's height (Popov, 1990) and would thus increase the structure's resistance to bending moments. This supports the idea that the morphology of the mandible at least has been moulded to support the high bite forces generated by parrots (Harrison et al., 2025). By contrast, the three specialised feeding modes of the Anseriformes suggested that mandible dimensions should scale isometrically in this order. Both body mass and mandible length, body mass and mandible height, were isometric which supports the idea that mandible dimensions in Anseriformes scale with body size and are not strongly influenced by factors such as diet and bite force. Moreover, birds of prey (Accipitriformes and Falconiformes) typically kill their prey with their talons (Sustaita, 2008) which helps explain their weak bite forces and small jaw musculature masses (Sustaita & Hertel, 2010), which are much lower for any given body mass than both the Psittaciformes and Anseriformes (Deeming et al., 2022, 2024). This suggests that the evolution of mandible morphology may not have been primarily driven by mechanical forces. To date, analysis of beak shape in these birds has been limited (Çakar et al., 2024; Pecsics et al., 2018) with no specific studies of the mandible, so further research could explore dimensions of the skull and mandible to test the prediction that mandible dimensions of birds of prey will scale with negative allometry with body mass.

The thinner shapes of mandibles were representative of smaller parrot species (<40 g) whereas species with more negative PC2 values, were much larger (>1000 g). Therefore, it could be that the larger parrots have a proportionately larger skull (represented by the wider cranial, maxilla and mandible shapes) which enables the ingestion of larger food items (Bright et al., 2016). This idea supports previous studies that showed that cranial and beak shapes were mainly reflective of body size in raptors (Bright et al., 2016), corvids (Kulemeyer et al., 2009) and pigeons (Young et al., 2017). In addition, larger finches (Fringillidae) tend to have more robust beaks (van der Meij & Bout, 2008). This association between skull shape and body size has also been demonstrated within mammalian families with smaller animals having significantly shorter facial forms compared to larger animals in the same family (Cardini & Polly, 2013). Similarly, in marsupials, the shape of the mandible was longer in insectivores, such as the Numbat (*Myrmecobius fasciatus*) which possessed a relatively thin and elongated jaw, whilst carnivorous species, such as the Western quoll (*Dasyurus geoffroii*), had relatively shorter mandibles (Morales‐García et al., 2021).

These observations coincide with the idea that whilst diet and beak shape are not necessarily as tightly confined as initially thought, the use of feeding structures, that is, bite force or object manipulation may be indicative of a specific diet (Homberger, 2003). Whilst the choice of food items has been found to be associated with a specific beak shape, implying that morphological constraints have a greater impact on diet choice, other factors may be involved. Hrabar and Perrin (2002) found that the Fisher's loverbird (*Agapornis fischeri*) consistently chose to feed on Japanese millet (*Echinochloa frumentacea*), despite being given the option of more nutritionally valuable food items. It was thought that this preference was regulated by the fact that Japanese millet was easier to handle by the bird due to its more elongate shape. Therefore, handling of a food item, which is largely influenced by the food's physical characteristic and how they aligned with the target species' jaw, appeared to influence overall food choice. This suggests that it may be that form is affecting function in the case of the avian beak, in that physical feeding morphology dictates the typical diet and not the other way around.

A new issue here is that it is perhaps difficult to make a clear association between body size and beak shape. It may be simply that the size of a bird and its beak may dictate the food items it could process, with bigger birds handling food items that require more processing. Bigger birds have more jaw musculature, which in turn corresponds with greater bite force (Deeming et al., 2022, 2024; Harrison et al., 2022, 2025). Therefore, any observed relationships may reflect an indirect association between body mass, skull size, jaw muscle mass and consequently bite force, rather than a direct effect of body size on cranial and beak shape. These features may be reflected in the analysis controlling for body mass, confounding interpretations of body mass and shape relationships. Whether this idea applies in parrots requires analysis of a much larger sample of jaw muscle masses and/or bite force values than is currently available (Carril et al., 2015; Cost et al., 2020).

The shape of the beak may also have been determined by factors not associated with the diet. For instance, beaks are used for preening and nest building (Sheard et al., 2023). Although beaks are used in locomotion in some birds (Lee & Walsh‐McGee, 1998; Vogel, 1950), in parrots, the beak is commonly used during locomotion and climbing (Young et al., 2022, 2023), as well as during beakiation locomotion along branches (Dickinson et al., 2024). It is possible that these kinds of activity have shaped the functional morphology of the parrot beak in ways so far unappreciated.

## 5 CONCLUSIONS

The aim of the study described in this report was to determine if there was a significant relationship between preferred diet and cranial and/or beak shape in parrots using geometric morphometrics. These differences were highly likely linked with body masses, which suggested that the morphology of the skull is influenced and conserved between different body sizes. Our results indicate that there is a significant link between beak shape and diet in parrots, but the level of influence the two factors have on one another is not as tightly defined as previously thought. These results lend support to the emerging idea that, whilst being an important influence on beak shape, diet is not the sole selective factor acting on beak shape. Inter‐clade variation in beak shape appears to be established early in evolutionary divergence and thereafter beak shape tends to be conserved in many, but not all, avian orders. Further study into functional aspects which are directly linked to the beak of parrots, for example bite force, is required to improve our understanding of the factors influencing avian cranial diversity.

## AUTHOR CONTRIBUTIONS

D.C.D., G.P.S. and S.L.H. conceived the ideas and designed methodology; S.L.H. collected the data; D.C.D., G.P.S. and S.L.H. analysed the data; D.C.D. led the writing of the manuscript. All authors contributed critically to the drafts and gave final approval for publication. S.L.H. and GPS were supported by grant UF120507 from the Royal Society.

## CONFLICT OF INTEREST STATEMENT

The authors declare no conflict of interest.

## Supporting information


Appendix S1.

## Data Availability

The data that support the findings of this study are available from the corresponding author upon reasonable request.
