# Supplementary material for: Fit for purpose? Analysis of the relationship between skull, beak shape and feeding ecology in Psittaciformes
Source: J Anat. 2025 Nov 4;248(5):873–87. doi: 10.1111/joa.70063 (PMC13069144; doi:10.1111/joa.70063)
Supplement: Supplementary file 1 — Appendix S1. [file JOA-248-873-s001.docx]

# **Fit for purpose? Analysis of the relationship between skull, beak shape and feeding ecology in Psittaciformes.**

Shannon L. Harrison, Gregory P. Sutton, & D. Charles Deeming

Department of Life Sciences, School of Natural Sciences, University of Lincoln, Joseph Banks Laboratories, Lincoln, LN6 7DL, UK

**Supplementary materials**

**Table S1**. List of species used in the maxilla, mandible, and cranial and maxilla analysis. 1 = present in sample, 0 = absent in sample. Three separate locations, Natural History Museum, Tring, <https://skullsite.com/>, and Lincolnshire Wildlife Park (LWP). provided the images of the skulls.

| Species | Photo location | Maxilla | Mandible | Cranial | Diet category |
| --- | --- | --- | --- | --- | --- |
| *Agapornis lilianae* | Skullsite | 1 | 1 | 1 | Herbivore |
| *Agapornis nigrigenis* | Skullsite | 1 | 0 | 1 | Seeds |
| *Agapornis personatus* | Tring | 1 | 0 | 1 | Seeds |
| *Agapornis pullarius* | Skullsite | 1 | 1 | 1 | Seeds |
| *Agapornis roseicollis* | Tring | 1 | 1 | 1 | Seeds |
| *Agapornis taranta* | Skullsite | 1 | 0 | 1 | Fruitseed |
| *Alisterus scapularis* | Skullsite | 1 | 1 | 1 | Herbivore |
| *Amazona aestiva* | Tring | 1 | 1 | 1 | Fruitseed |
| *Amazona albifrons* | Skullsite | 1 | 1 | 1 | Herbivore |
| *Amazona amazonica* | LWP | 1 | 1 | 1 | Fruit |
| *Amazona auropalliata* | LWP | 1 | 1 | 1 | Herbivore |
| *Amazona autumnalis* | LWP | 1 | 1 | 1 | Fruit |
| *Amazona barbadensis* | Skullsite | 1 | 1 | 1 | Herbivore |
| *Amazona dufresniana* | Skullsite | 1 | 1 | 1 | Fruitseed |
| *Amazona farinosa* | Tring | 1 | 1 | 1 | Fruit |
| *Amazona leucocephala* | Skullsite | 1 | 1 | 1 | Herbivore |
| *Amazona ochrocephala* | LWP | 1 | 1 | 1 | Herbivore |
| *Amazona oratrix* | LWP | 1 | 1 | 1 | Herbivore |
| *Amazona viridigenalis* | Skullsite | 1 | 1 | 1 | Herbivore |
| *Anodorhynchus hyacinthinus* | Tring | 1 | 1 | 1 | Fruit |
| *Aprosmictus erythropterus* | Tring | 1 | 1 | 1 | Omnivore |
| *Aprosmictus jonquillaceus* | Skullsite | 1 | 1 | 1 | Omnivore |
| *Ara ararauna* | Tring | 1 | 1 | 1 | Herbivore |
| *Ara chloropterus* | LWP | 1 | 1 | 1 | Herbivore |
| *Ara macao* | Tring | 1 | 1 | 1 | Herbivore |
| *Ara militaris* | Tring | 1 | 1 | 1 | Fruit |
| *Ara rubrogenys* | Skullsite | 1 | 1 | 1 | Herbivore |
| *Aratinga erythrogenys* | Skullsite | 1 | 1 | 1 | Fruit |
| *Aratinga pertinax* | Skullsite | 1 | 1 | 1 | Herbivore |
| *Aratinga solstitialis* | Tring | 1 | 1 | 1 | Fruit |
| *Aratinga wagleri* | Tring | 1 | 1 | 1 | Seeds |
| *Barnardius zonarius* | Tring | 1 | 1 | 1 | Omnivore |
| *Brotogeris cyanoptera* | Tring | 1 | 1 | 1 | Fruit |
| *Cacatua alba* | Tring | 1 | 1 | 1 | Omnivore |
| *Cacatua galerita* | Tring | 1 | 1 | 1 | Herbivore |
| *Cacatua goffiniana* | Skullsite | 1 | 1 | 1 | Herbivore |
| *Cacatua haematuropygia* | Skullsite | 1 | 1 | 1 | Fruitseed |
| *Cacatua leadbeateri* | Tring | 1 | 1 | 1 | Omnivore |
| *Cacatua moluccensis* | Tring | 1 | 1 | 1 | Fruitseed |
| *Cacatua roseicapilla* | Tring | 1 | 1 | 1 | Seeds |
| *Cacatua sulphurea* | Tring | 1 | 1 | 1 | Fruitseed |
| *Cacatua tenuirostris* | Skullsite | 1 | 1 | 1 | Seeds |
| *Calyptorhynchus funereus* | Tring | 1 | 1 | 1 | Omnivore |
| *Chalcopsitta duivenbodei* | Tring | 1 | 1 | 1 | Nectar |
| *Chalcopsitta sintillata* | Skullsite | 1 | 1 | 1 | Nectar |
| *Charmosyna josefinae* | Skullsite | 1 | 1 | 1 | Nectar |
| *Charmosyna papou* | Skullsite | 1 | 1 | 1 | Herbivore |
| *Coracopsis nigra* | Tring | 1 | 1 | 1 | Fruit |
| *Cyanoliseus patagonus* | Skullsite | 1 | 0 | 1 | Herbivore |
| *Cyanoramphus malherbi* | Skullsite | 1 | 1 | 1 | Fruit |
| *Deroptyus accipitrinus* | Skullsite | 1 | 1 | 1 | Herbivore |
| *Diopsittaca nobilis* | Skullsite | 1 | 0 | 1 | Fruitseed |
| *Eclectus roratus* | Skullsite | 1 | 1 | 1 | Herbivore |
| *Enicognathus ferrugineus* | Tring | 1 | 1 | 1 | Herbivore |
| *Enicognathus leptorhynchus* | Skullsite | 1 | 1 | 1 | Herbivore |
| *Eos cyanogenia* | Tring | 1 | 1 | 1 | Nectar |
| *Eos reticulata* | Skullsite | 1 | 1 | 1 | Seeds |
| *Eos squamata* | Skullsite | 1 | 1 | 1 | Omnivore |
| *Eunymphicus cornutus* | Skullsite | 1 | 0 | 1 | Nectar |
| *Forpus passerinus* | Skullsite | 1 | 1 | 1 | Herbivore |
| *Glossopsitta concinna* | Skullsite | 1 | 1 | 1 | Omnivore |
| *Guaruba guarouba* | Tring | 1 | 1 | 1 | Fruit |
| *Lathamus discolor* | Skullsite | 1 | 1 | 1 | Omnivore |
| *Loriculus vernalis* | Skullsite | 1 | 1 | 1 | Herbivore |
| *Lorius chlorocercus* | Skullsite | 1 | 1 | 1 | Omnivore |
| *Lorius garrulus* | Skullsite | 1 | 1 | 1 | Nectar |
| *Lorius lory* | Tring | 1 | 1 | 1 | Omnivore |
| *Melopsittacus undulatus* | LWP | 1 | 1 | 1 | Seeds |
| *Myiopsitta monachus* | Tring | 1 | 0 | 1 | Omnivore |
| *Nandayus nenday* | Tring | 1 | 1 | 1 | Seeds |
| *Neophema elegans* | Skullsite | 1 | 1 | 1 | Seeds |
| *Nestor notabilis* | Tring | 1 | 1 | 1 | Omnivore |
| *Nymphicus hollandicus* | Tring | 1 | 1 | 1 | Seeds |
| *Orthopsittaca manilata* | Skullsite | 1 | 1 | 1 | Fruit |
| *Phigys solitarius* | Tring | 1 | 1 | 1 | Nectar |
| *Pionites melanocephalus* | Tring | 1 | 1 | 1 | Nectar |
| *Pionus maximiliani* | Skullsite | 1 | 1 | 1 | Seeds |
| *Pionus senilis* | Tring | 1 | 1 | 1 | Fruitseed |
| *Pionus tumultuosus* | Skullsite | 1 | 1 | 1 | Fruit |
| *Platycercus adscitus* | Tring | 1 | 1 | 1 | Herbivore |
| *Platycercus eximius* | Tring | 1 | 0 | 1 | Omnivore |
| *Platycercus venustus* | Tring | 0 | 1 | 0 | Seeds |
| *Poicephalus cryptoxanthus* | Tring | 1 | 1 | 1 | Herbivore |
| *Poicephalus gulielmi* | Skullsite | 1 | 1 | 1 | Omnivore |
| *Poicephalus meyeri* | Skullsite | 1 | 1 | 1 | Omnivore |
| *Poicephalus robustus* | Tring | 1 | 1 | 1 | Herbivore |
| *Poicephalus rueppellii* | Skullsite | 1 | 1 | 1 | Omnivore |
| *Poicephalus rufiventris* | Skullsite | 1 | 0 | 1 | Fruitseed |
| *Poicephalus senegalus* | LWP | 1 | 1 | 1 | Herbivore |
| *Polytelis alexandrae* | Skullsite | 1 | 1 | 1 | Seeds |
| *Primolius maracana* | Tring | 1 | 1 | 1 | Seeds |
| *Probosciger aterrimus* | Tring | 1 | 1 | 1 | Herbivore |
| *Prosopeia tabuensis* | Tring | 1 | 1 | 1 | Fruit |
| *Psephotus varius* | Tring | 1 | 1 | 1 | Seeds |
| *Pseudeos fuscata* | Tring | 1 | 1 | 1 | Nectar |
| *Psittacula cyanocephala* | Skullsite | 1 | 1 | 1 | Fruit |
| *Psittacula derbiana* | Tring | 1 | 1 | 1 | Fruitseed |
| *Psittacula eupatria* | LWP | 1 | 1 | 1 | Herbivore |
| *Psittacula krameri* | LWP | 1 | 1 | 1 | Fruit |
| *Psittacula longicauda* | Tring | 1 | 1 | 1 | Fruitseed |
| *Psittaculirostris edwardsii* | Skullsite | 1 | 1 | 1 | Fruit |
| *Psittaculirostris salvadorii* | Tring | 1 | 1 | 1 | Fruit |
| *Psittacus erithacus* | Tring | 1 | 1 | 1 | Fruitseed |
| *Psitteuteles goldiei* | Tring | 1 | 1 | 1 | Nectar |
| *Psitteuteles iris* | Tring | 1 | 1 | 1 | Nectar |
| *Purpureicephalus spurius* | Tring | 1 | 1 | 1 | Omnivore |
| *Pyrilia pulchra* | Tring | 1 | 1 | 1 | Fruit |
| *Strigops habroptila* | Skullsite | 1 | 1 | 1 | Herbivore |
| *Tanygnathus megalorynchos* | Tring | 1 | 0 | 1 | Fruit |
| *Tanygnathus sumatranus* | Tring | 1 | 1 | 1 | Fruit |
| *Trichoglossus euteles* | Skullsite | 1 | 0 | 1 | Nectar |
| *Trichoglossus haematodus* | Tring | 1 | 1 | 1 | Herbivore |
| *Vini peruviana* | Tring | 1 | 1 | 1 | Nectar |
| *Vini ultramarina* | Tring | 1 | 1 | 1 | Nectar |


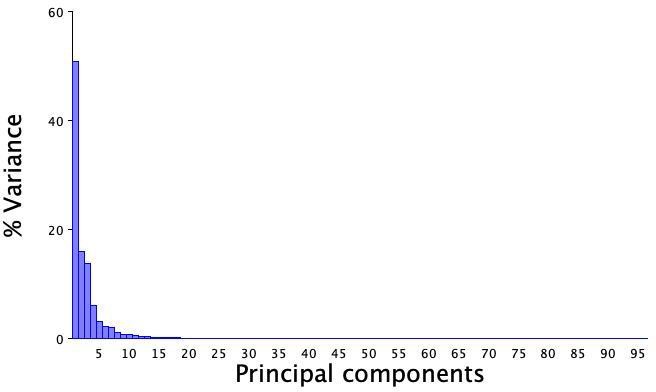

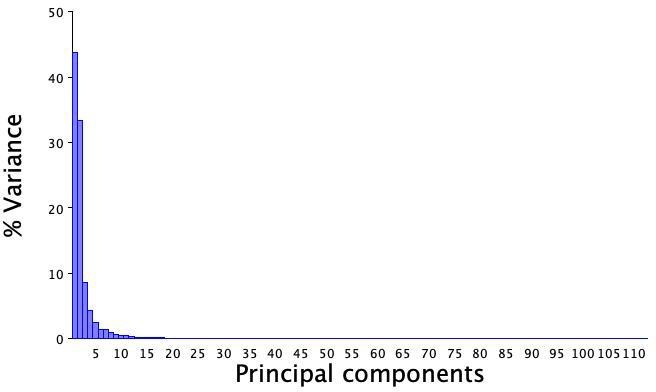

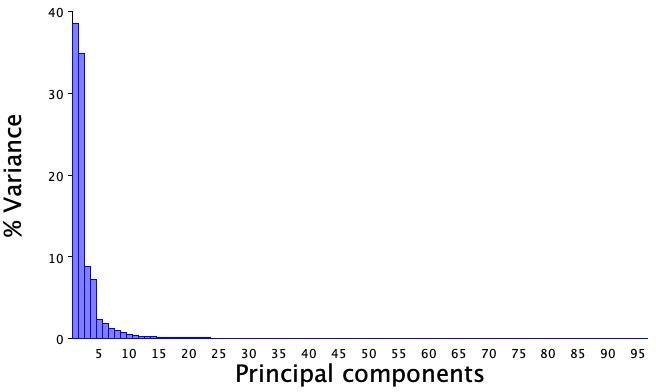


**Figure S1**. Scree plots of percentage variance explained by each PC score for the maxilla (top panel), lower mandible (middle panel), and cranial and maxilla shape difference (lower panel).

Figure S2(a) Phylogeny of species used in both the Upper beak and cranial analyses. Downloaded from Birdtree.org (Jetz *et al*., 2014).

Figure S2(b) Phylogeny of species used in the mandible analyses. Downloaded from Birdtree.org (Jetz *et al*., 2014).
